# Histological, ultrastructural and biochemical studies on the kidney of mice treated with Carthamus tinctorius L. extract

**Published:** 2013

**Authors:** Ali Louei Monfared

**Affiliations:** 1 ***Department of Basic Sciences, Faculty of Para-Veterinary Medicine, University of Ilam, Ilam, I. R. Iran***

**Keywords:** *Carthamus tinctorius*, Electron microscopy, Histology, Kidney, Mice

## Abstract

**Objective:** *Carthamus tinctorius* L. (*C. tinctorius*) is used as a food additive but also has medicinal applications. The present work was designed to investigate its probable side effects on the histology and function of the kidney in the mice.

**Materials and Methods: **Sixty adult Balb/C mice were randomly distributed into one control and three experimental groups. The control group received only distilled water, while experimental groups were administered intraperitoneally* C. tinctorius* at doses of 0.7, 1.4, and 2.8 mg/kg for 49 consecutive days. In the end of experiments after blood sampling, the biochemical analyses of plasma were performed. Tissue samples were also taken and structural alterations were examined using light and electron microscopes.

**Results: **There were histological changes included decreasing in the diameter of glomerules, increase of proximal tubular lumen, tubular necrosis, leuckocyte infiltration, and massive congestion in the kidney of the 1.4 and 2.8 mg/kg* C. tinctorius* groups. Moreover, ultrastructural study revealed destruction of the glomerular basement membrane, shrinkage of podocyte΄s nucleus, and reduction in the number and size of microvilli in epithelial cells of renal tubules. Furthermore, the levels of creatinine in the plasma of 1.4 and 2.8 mg/kg* C. tinctorius* groups showed a significant increase in comparison with the control group (p<0.05).

**Conclusion: **It is concluded that *C. tinctorius* extract exposure at doses of 1.4 and 2.8 mg/kg has harmful effects on the renal tissue and therefore, popular consumption of this plant should be reconsidered.

## Introduction

Herbal medicine is a complementary therapy that uses plants to treat disorders. In various countries throughout the world, a large number of plants have been used as therapeutic agents in the traditional medicine (Kumar et al., 2012 ▶), but there are no enough documents in the literature about their probable toxic effects. 

*Carthamus tinctorius* (Safflower) is a plant species of asteraceae family (Siddiqi et al., 2009 ▶) which its flowers have applications in medical settings and food industry (Elias et al. 2002 ▶; Mass 1986 ▶). For instance, it has been shown that the concentration of 31.25, 62.5, and 125 μg/mL of plant extract possesses the ability to suppress JNK activity and inhibit LPS-induced TNFα activation and apoptosis in H9c2 cardiomyoblast cells (Tien et al., 2010). In contrast, there are many studies in the literature indicate the side effects of *C. tinctorius* extract administration in the laboratory animals. For instance, our previous works documented the developmental toxic impacts of *C. tinctorius *treatment on the mice embryo during organogenesis period (Louei Monfared et al., 2012 ▶). 

Another study demonstrated the adverse effects of safflower extract administration on the placental histomorphology and survival of mice neonates (Louei Monfared and salati, 2012 ▶). In that study, *C. tinctorius* extract at doses of 1.4 and 2.8 mg/kg could induce toxic alterations in the placental structure and also significantly decreased survival of the neonates. In addition, Nobakht et al. (2000 ▶) demonstrated an association between maternal exposure to *C. tinctorius* extract and occurrence of congenital malformations in their offspring. Furthermore, Mirhoseini et al. (2012) ▶ showed the toxic effects of *C. tinctorius* extract on the mouse spermatogenesis and testicular tissue alterations. These authors attributed the toxic effects of *C. tinctorius* extract to the action of vasodilator substances such as serotonin which exist in the plant structure. 

Since there is very little information about the effects of *C. tinctorius* on the histology and function of the kidney, this study was done.

## Materials and Methods

*Carthamus tinctorius* (Safflower or Golrang) plants were purchased from Emam-Reza medicinal plants market (Ilam, Iran) and botanical identification was confirmed at the herbarium of Ilam University (Herbarium Number: 135-6-91). For extract preparation, the plant material was washed with sterile water, dried in shade at room temperature for two weeks and ground in an electric mill until particles less than 4 mm were obtained. This material was extracted by maceration in 70% methanol solution at 50 ºC during 2 hours. The extract was filtered through a Wattman ≠1 paper and evaporated to dryness in a rotary evaporator under reduced pressure. The dried material was stored under refrigeration at 4-8 ºC until its use. 

A total of sixty adult female Balb/C mice at 28±9 grams of initial body weight and aged ten weeks were purchased from Razi Institute (Karaj, Iran). The animals were housed in a controlled environment (temperature of 23 ± 1 ºC; relative humidity 45±5%; 12:12 h light-dark natural cycle) and had ad-lib access to drinking water and food. Mice were allowed to be acclimatized to the laboratory environment at least one week before commencement of testing. Animals were randomly distributed into one control and three experimental groups (n= 15). The control group received only distilled water, while experimental groups were administered intraperitoneally *C. tinctorius* extract at doses of 0.7, 1.4, and 2.8 mg/kg once a day for 49 consecutive days. The doses were determined on the basis of a primary study. 

At the end of experiments, the animals were weighted and anesthetized. Then, the blood samples were collected via direct cardiac puncture. Plasma was separated by centrifugation at 2500 rpm for 15 minutes and stored at -20 ºC until analysis. Plasma samples were analyzed for total protein (TP) by the Biuret method, creatinine (CRT) according to Jaffe method, blood urea nitrogen (BUN) by the modified urease-Berthelot method, and alkaline phosphatase (ALP) activity by enzymatic (IFCC) method, using a spectrophotometer (Shimadzu, Model AA200, Tokyo, Japan). Commercial colorimetric kits were obtained from Pars Azmun Co. (Tehran, Iran).

For light microscopy study, left kidney immersion imprisoned overnight in 10% neutral buffered formalin to be fixed. Then the kidneys were mounted to allow 5-µm sections. They were stained via hematoxylin and eosin (H&E). Sections were photographed directly using a light microscope in 400 high power fields. Photographs were taken with a digital camera (COOLPIX 950, Nikon, China) and stored. Then, the diameter of glomerulus, diameters of urinary space, proximal, and distal tubular lumen were determined by Image Tool® 3.0 software (UTHSCSA, San Antonio, TX, USA) and compared between experimental and control groups. 

For electron microscopy, small pieces (2 mm) of the left kidney were fixed in 2.5% glutaraldehyde. The specimens were put in 0.1 M phosphate buffer (pH = 7.3) at 4 ºC for 4 hours. After washing twice (30 min each) with cold 0.2 M phosphate buffer, the tissues were post-fixed in 1% osmium tetroxide at 4 ºC for 4 hours. The specimens were dehydrated in gradual series of ethanol and embedded in Epon 812. Ultra thin sections (600 Å) were cut employing an ultra microtome (Ultracut, Reichert-Jung, Austria), mounted onto copper grids and stained with uranyl acetate and subsequently with lead citrate (Reynolds, 1963 ▶). The grids were examined using Zeiss 902 electron microscope.

All quantitative data were expressed as standard error of the mean (SEM). The analysis of variance (ANOVA) was used to test the overall significance of differences among the means. Tukey-Kramer´s Multiple Comparison test was applied for *post-hoc* comparison. Computations were performed using site-licensed SPSS statistical software (SPSS, Chicago, IL, USA). A probability level of less than 5% (p<0.05) was considered as significant. 

## Results

**Figure 1:**
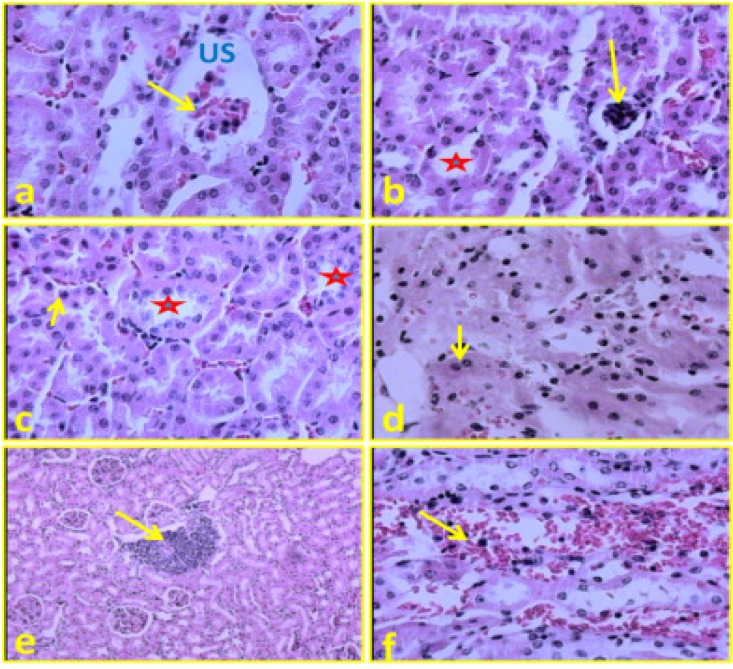
a). Kidney transverse section of the treated mice with C. tinctorius at the concentrations of 1.4 mg/kg/day. The section shows decreasing in the diameter of glomerules (arrow) and increasing in urinary space (US). (b): Kidney transverse sections of the mice treated with C. tinctorius at the concentrations of 2.8 mg/kg/day. The section shows decreasing in the diameter of glomerules (arrow) and increase of proximal tubular lumen (star). (c): Kidney transverse section of the treated mice with C. tinctorius at the concentrations of 1.4 mg/kg/day. The section shows tubular necrosis in the epithelial cells of renal tubules (arrow) and increase of proximal tubular lumen (star). (d): Kidney transverse sections of the mice treated with C. tinctorius at the concentrations of 2.8 mg/kg/day. The section shows tubular necrosis in the epithelial cells of renal tubules (arrow). (e): Kidney transverse section of the treated mice with C. tinctorius at the concentrations of 1.4 mg/kg/day. The section shows severe leuckocyte infiltration (arrows). (f): Kidney transverse sections of the mice treated with C. tinctorius at the concentrations of 2.8 mg/kg/day, showing massive congestion in the renal parenchyma (arrow). (Haematoxylin and Eosine stain) (a,b,c,d, and f: ×400 , e: ×100).

In the *C. tinctorius* extract-treated animals at doses of 0.7 mg/kg there was not any outstanding finding in the structural or functional integrity of kidney when compared with the control group. Although, in the kidney of animals administrated with 1.4 and 2.8 mg/kg* C. tinctorius*, there were many histological alterations included decreasing in the diameter of glomerules, increasing in urinary space, increasing of proximal tubular lumen as well as tubular necrosis, leuckocyte infiltrationand, and also massive congestion (Figure 1 and Table 1). 

**Table 1:** Mean±SEM of the histometric parameters of the kidney in the control and treated mice with different doses of *C. tinctorius* extract (n = 15).

Renal Parameters/Groups	Control	0.7 mg/kg/day *C. tinctorius*	1.4 mg/kg/day *C. tinctorius*	2.8 mg/kg/day *C. tinctorius*
**Diameter of Glomerulus (µm)**	125.9 ± 6.3	124.62 ± 3.7	58.9 ± 2.7^*^	53.5 ± 8.4^*^
**Diameter of Urinary Space (µm)**	4.6 ± 0.77	4.7 ± 1.3	9.4 ± 0.6^*^	11.8 ± 3.5 ^*^
**Diameter of Proximal Tubular Lumen (µm)**	10.7 ± 0.5	13.2 ± 0.6	43.6 ± 1.59 ^*^	43.54 ± 2.72 ^*^

* **Significant differences with the control group as p<0.05.**

**Figure 2:**
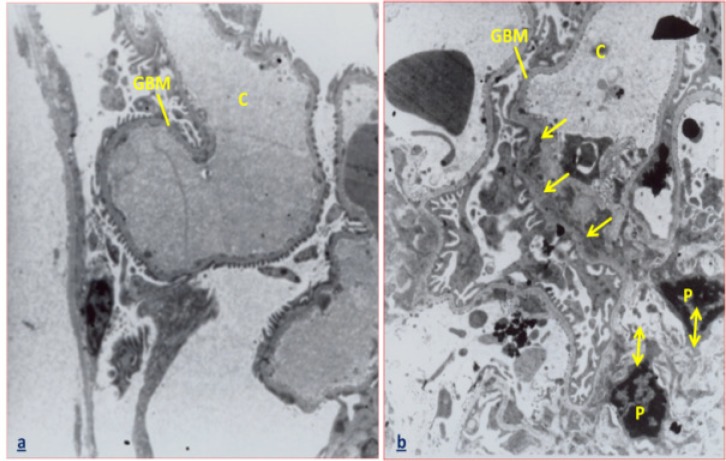
(a): Electron micrograph of a part of kidney in the control mice showing the normal structure of the glomerulus.(b): Electron micrograph of a part of kidney in the treated mice with C. tinctorius at a concentration of 1.4 mg/kg/day showing a part of glomerulus. The arrows indicate that the glomerular basement membrane (GBM) is destructed and un-continues. Moreover, double-head arrows reveal the shrinkage of podocyte΄s nucleus in the glomerulus. C: capillary of glomerulus, GBM: glomerular basement membrane, P: podocyte΄s nucleus. (×3000).

**Figure 3:**
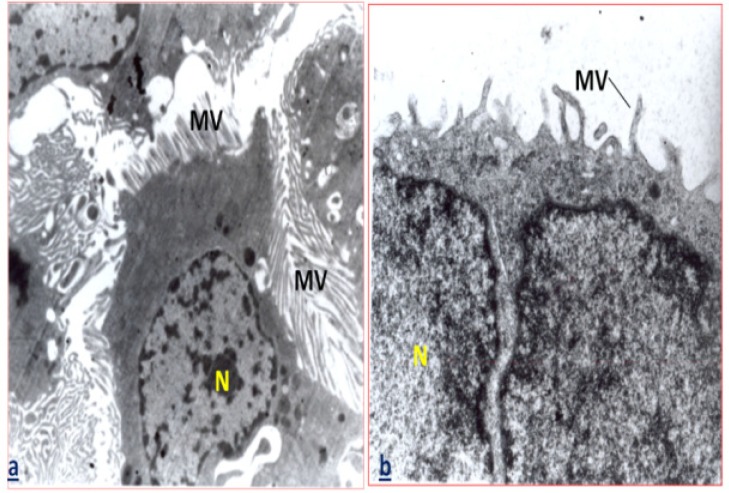
(a): Electron micrograph of a part of kidney in the control mice showing the normal number and size of apical microvilli in the epithelial cells of proximal convoluted tubules. (b): Electron micrograph of a part of kidney in the treated mice with C. tinctorius at the concentrations of 2.8 mg/kg/day, showing a part of lining cells of proximal convoluted tubules. This micrograph shows a nucleus with decreasing in the number and size of apical microvilli in epithelial cells of proximal convoluted tubules. MV: microvilli, N: nucleus (×7000).

Moreover, ultra structural study revealed that treatment with 1.4 and 2.8 mg/kg of *C. tinctorius* could induce destruction of the glomerular basement membrane, shrinkage of podocyte΄s nucleus (Figure 2), and reduction in the number and size of microvilli in epithelial cells of proximal convoluted tubules (Figure 3). Furthermore, the plasma levels of creatinine and also the activity of ALP in the animals administrated with 1.4 and 2.8 mg/kg* C. tinctorius* revealed a significant increase in comparison with those of the control group (p<0.05, Table 2).

**Table 2:** Mean±SEM of the plasma levels of renal function parameters in the control and treated mice with different doses of C. tinctorius extract (n = 15).

Renal Performance Parameters/Groups	Control	0.7 mg/kg/day *C. tinctorius*	1.4 mg/kg/day *C. tinctorius*	2.8 mg/kg/day *C. tinctorius*
**Total Protein (g/dL)**	3.87 ± 0.37	3.73 ± 0.5	3.68 ± 0.12	3.89 ± 0.84
**CRT (mg/dL)**	0.34 ± 0.08	0.36 ± 0.08	1.06 ± 0.09^*^	1.28 ± 0.05^*^
**BUN (mg/dL)**	30.47 ± 1.47	33.3 ± 1.67	53.6 ± 1.79 ^*^	47.1 ± 3.9 ^*^
**ALP (U/L)**	59.29 ± 3.18	53.7 ± 2.86	120.3 ± 6.24 ^*^	126.7 ± 3.73^*^

* Significant differences with the control group as p<0.05.

## Discussion

It has been reported that *C. tinctorius* has immense medicinal and therapeutic properties (Bahmanpour et al., 2012 ▶). It has been demonstrated that herbal toxicity clearly represents a serious human health threat and is an important issue to be tackled (Chen et al., 2011 ▶). Moreover, herbs have a variety of complex chemical constituents that act on the body as a whole or on specific organ and systems. Some of the chemical constituents are mild and safe even in large doses while, some act more strongly or toxic in large doses or when taken continuously (Chen et al., 2011 ▶).

Our results showed that both histological and functional parameters of the kidney were altered after treatment with *C. tinctorius *extract at doses of 1.4 and 2.8 mg/kg/day. Therefore, the components of the extract of this plant can induce harmful effects on the renal tissue. In contrast, previous work demonstrated that the concentrations of 31.25, 62.5, and 125 μg/mL of plant extract possess the ability to suppress JNK activity and inhibit LPS-induced TNFα activation and apoptosis in H9c2 cardiomyoblast cells (Tien et al., 2010). This dissimilarity may be associated with the differences in the extract concentrations between the two studies. 

In the current work, the necrotic alteration in the epithelium of renal tubules under light microscope is supported by loss of microvilli in epithelial cells of proximal convoluted tubules under electron microscopic assay. Massive congestion was found in the renal parenchyma of the 1.4 and 2.8 mg/kg/day* C. tinctorius *extract-administrated animals is in accordance with previous reported vascular dilation and congestion of the testis after treatment with this plant (Mirhoseini et al., 2012 ▶). These changes could be a consequence of the action of vasodilator substances such as serotonin which is present in the *C. tinctorius* extract (Suzuki et al., 2010 ▶).

Dilation of urinary space of renal corpuscle in the 1.4 and 2.8 mg/kg/day* C. tinctorius *extract-treated mice may be due to basement membrane alterations and proximal convoluted tubules epithelium changes such that decreasing the functional properties of renal tubules, resulted in GFR decreasing and accumulation of urine in the urinary space. It is documented that normality of the basement membrane of glomerulus is essential for normal glomerular filtration rate (GFR) and any change in this structure leads to proteinuria (Nakamura et al., 2004 ▶).

Estimation of renal excretion of waste metabolites and histological changes in the kidney has provided useful information on the health status of the kidneys (Panda, 1989 ▶). BUN is the waste product of protein metabolism which has to be excreted by the kidney. Therefore, it is found that the increases of CRT and BUN in this study indicate the biochemical damage to the kidney after treatment with *C. tinctorius *extract at doses of 1.4 and 2.8 mg/kg/day (Panda, 1989 ▶). Significant increasing in the plasma ALP activity in the present study may be due to cytotoxicity and it is observed in the proximal tubule cells when exposed to phenol (Tootian et al., 2012 ▶).

There are no exact mechanism(s) of action for *C. tinctorius* renal histological alterations in the literature. However, it has been reported that *C. tinctorius *plant has a variety of complex chemical constituents including flavonoids, glucosides, and rutinosides that could act on the body as a whole or on specific organs (Chen et al., 2011 ▶; Li Fan et al., 2009 ▶). Further investigations are needed to elucidate exact causative factors for *C. tinctorius*-induced renal structural alterations. 

Upon these findings, it is concluded that *C. tinctorius* extract exposure has harmful effects on the renal tissue and therefore popular consumption of this plant should be reconsidered.
